# Islamic mindfulness as cultural mindfulness: a conceptual framework for decision-making and well-being

**DOI:** 10.3389/fpsyg.2026.1715750

**Published:** 2026-02-11

**Authors:** Sadiq, Muhammad Salman Ahmad

**Affiliations:** 1 School of Business, Shandong Xiehe University, Jinan, Shandong, China; 2 School of Economics and Management, Yango University, Fuzhou, Fujian, China

**Keywords:** cultural specificity in mindfulness, decision-making, Islamic mindfulness, mindful consumption, workplace well-being

## Abstract

**Objectives:**

This conceptual study examines how Islamic mindfulness practices (*Muraqabah*, *Dhikr*, *Salah*) influence psychological processes that extend beyond spiritual benefits, shaping decision-making and well-being in both personal and professional domains.

**Methods:**

Employing a conceptual analysis methodology, this research synthesizes interdisciplinary literature from Islamic studies, psychology, consumer behavior, and organizational management to theoretically explicate the psychological mechanisms of these practices.

**Results:**

The analysis suggests that these practices may cultivate heightened self-regulation, intentionality (Niyyah), and ethical attentional orientations, including tendencies toward reduced materialism. This psychological profile is theorized to be associated with more deliberate and ethically oriented consumption tendencies and with orientations supporting professional resilience, focus, and ethical decision-making.

**Conclusion:**

This paper theorizes that Islamic mindfulness may provide a framework for understanding culturally embedded forms of mindfulness. While explicitly conceptual and exploratory in nature, it highlights directions for empirical validation and introduces a Culturally Embedded Mindfulness Model (CEMM) that contributes to ongoing debates in mindfulness science regarding universality versus cultural specificity.

## 1 Introduction

The global Muslim population, exceeding 1.9 billion individuals (Abu Khait and Lazenby, 2021), represents one of the most significant and dynamic consumer segments in the world economy. With an Islamic economy encompassing finance, food, lifestyle, and travel valued in the trillions of dollars (Qadri and Malik, 2024), this demographic’s purchasing power and cultural influence are undeniable (Kaya and Kaya, 2024). Academic and commercial inquiry into this market has often focused on the obvious markers of religious compliance, most notably the demand for *Halal* products permissible under Islamic law (Rahman et al., 2024). This external focus risks overlooking the deeper psychological and spiritual frameworks that shape Muslim consumers’ behavior and professional ethos. Beyond market size, however, understanding Muslim engagement in economic and professional life requires attention to the culturally embedded cognitive and ethical frameworks that shape everyday decision-making.

Concurrently, the secular field of mindfulness studies has burgeoned, establishing mindfulness commonly defined as the non-judgmental awareness of the present moment (Brown et al., 2007; Chems-Maarif et al., 2025) as a critical factor in enhancing mental health, emotional regulation, and cognitive performance. More recently, this research has expanded into the domains of consumer psychology and organizational behavior, revealing that mindfulness can reduce impulsive buying, promote sustainable consumption, increase job satisfaction, and mitigate burnout (Choi et al., 2022; Gupta and Sheth, 2024; Kumar et al., 2024). Typically, secular mindfulness is presented in acontextual, often Western-centric frameworks such as Mindfulness-Based Stress Reduction (MBSR).

A growing body of scholarship has questioned the cultural neutrality of mainstream mindfulness frameworks and emphasized the importance of situating mindfulness within diverse cultural, philosophical, and contemplative traditions. Cross-cultural perspectives highlight that mindfulness and related contemplative practices are historically embedded in specific ethical, religious, and cosmological systems, rather than being universally uniform psychological techniques (Christopher et al., 2009; Kitayama and Uskul, 2011; Lomas, 2019).

While Islamic mindfulness shares certain functional similarities with secular mindfulness, particularly in attentional anchoring and emotional regulation, it is grounded in distinct theological and structural foundations (Kabat-Zinn, 2003; Purser and Milillo, 2015). Central to Islamic mindfulness is transcendental accountability (*Muraqabah*), whereby awareness is framed as a continuous moral responsibility before God, rather than as value-neutral observation (Rothman and Coyle, 2018; Ghazali, 2023). The concept of *Niyyah* further embeds theological intentionality, requiring conscious alignment of actions with spiritual purpose prior to behavior, a principle rooted in foundational Islamic jurisprudence and prophetic tradition (Al-Bukhari, 1994; Kamali, 2003). Moreover, core practices such as Salah are obligatory, ritualized, and temporally structured, distinguishing Islamic mindfulness from the voluntary, individualized, and therapeutic orientation characteristic of most secular mindfulness approaches (Nasr, 2009; Rothman, 2021).

What remains a significant gap in the literature is the limited exploration of deeply culturally and religiously embedded mindfulness traditions and their specific impacts on secular economic activities (Henning et al., 2025). Within an Islamic context, mindfulness is not a contemporary therapeutic import but a centuries-old core of spiritual practice, intrinsically woven into the daily rituals and ethical fabric of a Muslim’s life (Aldbyani, 2025). This practice, known as *Muraqabah*, is cultivated through prescribed acts of worship such as the five daily prayers (*Salah*), the remembrance of God (*Dhikr*), and the foundational concept of conscious intention (*Niyyah*).

It is important to acknowledge, however, that Islamic mindfulness is not uniform or monolithic. While foundational concepts such as *Muraqabah*, *Salah*, *Dhikr*, and *Niyyah* are shared across the Islamic tradition, their interpretation, emphasis, and everyday enactment vary across regions, jurisprudential schools, and denominational traditions, including the Sunni school of thought and Shia school of thought (Kamali, 2003; Nasr, 2009). Cultural histories, local religious norms, and socio-political contexts further shape how these practices are understood and lived, suggesting that Islamic mindfulness is best conceptualized as a contextually embedded and heterogeneous orientation, rather than a singular or universal model (Rothman and Coyle, 2018).

A growing body of research has documented the benefits of these practices for mental health and spiritual well-being among Muslim populations (Ijaz et al., 2017; Purwanto et al., 2023; Aldbyani, 2025), yet their downstream effects on everyday choices and professional functioning have been largely neglected. Situating Islamic mindfulness within the broader mindfulness literature, therefore, provides a valuable opportunity to engage directly with debates on cultural specificity and universality in mindfulness science.

This paper aims to bridge this gap. It argues that the Islamic mindfulness framework actively and systematically shapes the economic and professional lives of its adherents. By consistently cultivating intentionality, self-regulation, and a focus on transcendental accountability, we theorize that Islamic mindfulness practices may contribute to a psychological profile predisposed toward deliberate consumption, ethical financial orientations, and resilient, focused professional performance. This analysis does not seek to reduce spiritual practice to mere economic utility, but to illuminate how a spiritually oriented cognitive framework manifests in and influences worldly domains.

To this end, this conceptual study has three primary objectives: first, to elucidate the principles and practices of Islamic mindfulness (*Muraqabah*) and its psychological consequences; second, to theorize the mechanisms through which these consequences influence consumer decision-making and workplace behavior; and third, to discuss the significant implications for marketers, organizational leaders, and future interdisciplinary research. In this conceptual analysis, consumer behavior and workplace well-being are examined as illustrative domains to demonstrate the broader applicability of the Culturally Embedded Mindfulness Model (CEMM), rather than as exhaustive or exclusive outcome contexts. This study contributes to a more nuanced and respectful understanding of Islamic mindfulness as a cultural system that shapes self-regulation, decision-making, and well-being.

As a conceptual study, this paper draws on a structured synthesis of interdisciplinary literature rather than original empirical data. Relevant studies were identified through targeted searches of major academic databases, including Web of Science, Scopus, and Google Scholar, with a focus on peer-reviewed research and authoritative scholarly works on mindfulness, Islamic psychology, religious practice, consumer behavior, and workplace well-being. Sources were selected for their conceptual relevance and theoretical contribution, and key themes were synthesized by tracing recurring constructs and linkages across these literatures, which informed the development of the Culturally Embedded Mindfulness Model (CEMM).

**FIGURE 1:**
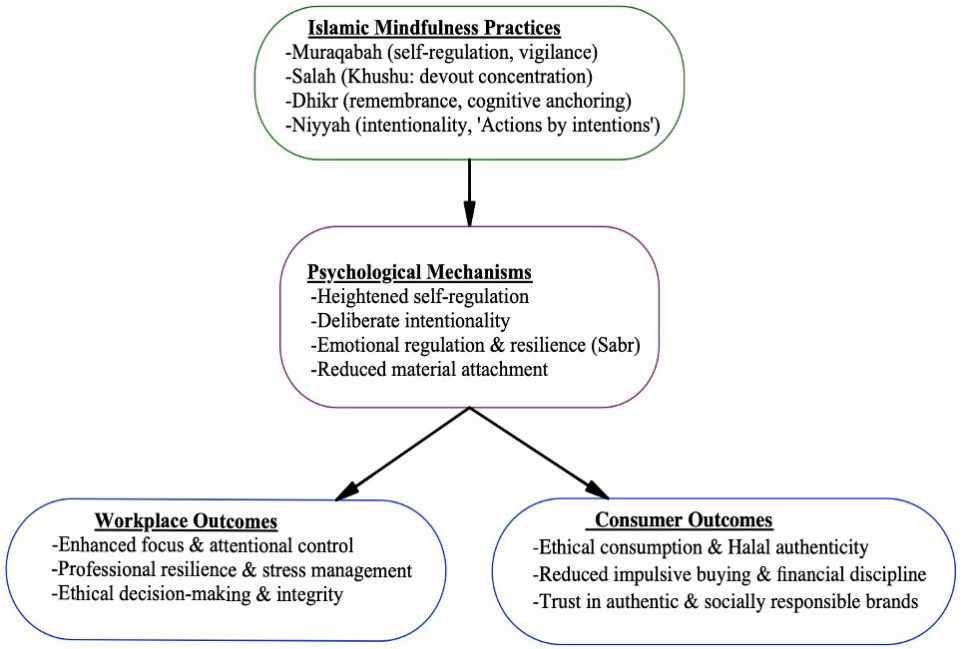
The Culturally Embedded Mindfulness Model (CEMM) is presented in a sequential form for conceptual clarity, while acknowledging context-dependent and potentially recursive relationships. Islamic mindfulness practices (*Salah*, *Dhikr*, and *Niyyah*) are theorized to cultivate psychological mechanisms such as intentionality, self-regulation, and moral self-monitoring, which in turn influence behavioral outcomes in consumption and workplace contents. Contextual factors including religiosity, cultural setting, and socioeconomic conditions may moderate these relationships.

Figure 1 presents the proposed Culturally Embedded Mindfulness Model (CEMM) to guide the discussion. It maps the relationship between Islamic mindfulness practices, their psychological mechanisms, and behavioral outcomes across personal and professional domains.

Conceptually, the Culturally Embedded Mindfulness Model (CEMM) proposes a sequential process in which Islamic mindfulness practices operate as antecedent conditions that activate internal psychological mechanisms, which subsequently shape behavioral outcomes. Specifically, practices such as *Salah*, *Dhikr*, and *Niyyah* cultivate sustained attentional awareness, intentional self-regulation, and moral self-monitoring (*Muraqabah*). These psychological mechanisms then influence downstream behaviors, including ethical consumption choices and work-related self-discipline. Accordingly, CEMM does not assume a direct behavioral effect of religious practice, but rather a mediated pathway through core psychological processes.

The strength and expression of these pathways may vary across individuals and contexts. Factors such as individual religiosity, cultural embeddedness, and socioeconomic conditions are likely to condition how strongly Islamic mindfulness practices translate into psychological mechanisms and observable behaviors. These factors are therefore conceptualized as contextual moderators, positioning CEMM as a culturally grounded framework rather than a universal behavioral model.

## 2 Theoretical foundations: deconstructing Islamic mindfulness (Muraqabah)

To understand the impact of Islamic mindfulness on behavior, one must first appreciate its theological and practical foundations. The term *Muraqabah* is derived from the Arabic root *ra-qaf-ba*, meaning watching, observing, guarding, and caring. In an Islamic context, *Muraqabah* describes a state of constant, vigilant awareness that one is being observed by Allah (SWT) in all actions, thoughts, and intentions (Nasir, 2012). It is the practical implementation of the Quranic concept that Allah (SWT) is ever-present and all-knowing: “*And indeed, We have created man, and We know what his soul whispers to him. And We are closer to him than his jugular vein*” (Abdel-Haleem, 2004, 50:16). This is not a mindfulness of emptiness, but a mindfulness of presence, a profound consciousness of a transcendent witness.

This awareness is not left as an abstract ideal but is embodied through a set of daily practices that function as structured mindfulness training. Within Islamic tradition, these practices are not peripheral; they are central mechanisms through which spiritual consciousness is continuously reinforced in ordinary life. Three such practices, Salah (ritual prayer), Dhikr (remembrance of God), and Niyyah (intention), represent the most salient and enduring forms of this operationalized mindfulness. Each practice fulfills religious obligations and simultaneously serves as a cognitive-ethical exercise that aligns with contemporary understandings of attentional control, emotional regulation, and intentionality.

### 2.1 Salah as mindful presence

Among the daily practices, *Salah* (the five obligatory prayers) is the most structured and immersive expression of mindfulness within Islamic practice. Its ritualized physical movements and recitation of Qur’anic verses demand complete cognitive and physical engagement, with distraction regarded as a spiritual and practical deficiency. The Prophet Muhammad (PBUH) is reported to have said, “*A servant may pray and have nothing recorded for it except a tenth of it, a ninth, eighth, seventh, sixth, fifth, quarter, third, or half of it*” (Abu Dawud, 2008). This underscores the requirement for *Khushu*, a state of devout concentration, humility, and heartfelt presence.

From a psychological perspective, this act of focused attention interrupts the automatic pilot of daily life, serving as a repeated exercise in attentional anchoring and self-regulation. Empirical studies by Ijaz et al. (2017) and Ahmed and Yousaf (2025) indicated that Muslims who engage in prayer with higher levels of mindful awareness report better psychological well-being, suggesting a link between the qualitative experience of prayer and psychological outcomes. This practice parallels attentional anchoring described in secular mindfulness interventions such as Mindfulness-Based Stress Reduction (MBSR), indicating functional similarities across cultural traditions (de Vibe et al., 2017).

### 2.2 Dhikr as cognitive anchoring

Complementing *Salah*, *Dhikr* (the remembrance of God) offers a more fluid practice of mindfulness that extends beyond fixed prayer times. *Dhikr* functions as a continual attentional anchor by repeating sacred phrases throughout daily routines, redirecting awareness toward transcendence amid ordinary activity. This can be formal, such as after prayers, or informal, woven throughout the day during mundane activities like driving, working, or waiting. Common *Dhikr* include *Subhan Allah* (Glory be to God), *Alhamdulillah* (All praise is due to God), and *Allahu Akbar* (God is the Greatest) (Aldbyani, 2025).

Much like the breath in secular mindfulness meditation, *Dhikr* continuously draws the individual’s awareness back from distracting thoughts to a transcendent focal point. Research by Purwanto et al. (2023) has shown that *Dhikr*-based breathing therapy is associated with improvements in quality of life among individuals with insomnia, while studies by Rahman et al. (2023) report increased feelings of gratitude and emotional balance. These findings suggest that Dhikr may support emotional regulation and reduced reactivity, aligning with mechanisms identified in secular mindfulness practices. Like mantra repetition in contemporary programs (Oman et al., 2025), Dhikr provides a cognitive focal point, bridging Islamic and modern mindfulness constructs.

### 2.3 Niyyah as intentionality

Finally, *Niyyah* (intention) frames every act of worship and daily conduct with conscious purpose. More than a legal requirement, it serves as a spiritual discipline that trains individuals to pause, reflect, and clarify their motivations before engaging in any action. In Islamic jurisprudence, every act of worship is invalid without a correct intention. However, the spiritual application extends far beyond ritual. The Prophet Muhammad (PBUH) is reported to have said, “*Indeed, actions are but by intentions, and every person shall have only that which they intended*” (Siddiqi, 1971; Al-Bukhari, 1994).

This doctrine instills a habit of pausing before an action, whether praying, eating, working, or purchasing, to articulate its purpose consciously. Is it for the pleasure of God? For personal necessity? For showing off? (Gupta and Sheth, 2024) This practice of pre-action reflection constitutes a formalized mechanism for cultivating intentionality and self-monitoring. While this aligns with goal-directed awareness in mindfulness research (Marion-Jetten et al., 2022), it introduces a theological orientation absent in secular frameworks.

Together, these practices cultivate a profile of self-regulation, deliberate intentionality, and ethically oriented attentional awareness, which conceptually links spiritual practice to broader behavioral orientations. This cultivated profile provides the theoretical bridge to subsequent discussions of consumer decision-making and workplace well-being, without presuming direct or deterministic behavioral effects.

## 3 Application of Islamic mindfulness

The psychological traits cultivated through *Muraqabah*, *Salah*, and *Dhikr* extend beyond the mosque or prayer mat, shaping patterns of everyday decision-making. This section explores how these practices influence deliberate and ethical choices, conceptually framed through three interlocking mechanisms.

### 3.1 Consumer decision-making

The first mechanism is the infusion of deliberate intentionality (*Niyyah*) into the consumption process. The habitual practice of formulating intention acts as a conceptual cognitive circuit breaker against impulsive consumption. The mindful Muslim consumer is theoretically encouraged to move beyond the initial desire for a product and interrogate its purpose and alignment with higher-order values (Floren et al., 2020). This involves asking questions such as: “Is this purchase necessary?” “Does it align with my role as a responsible steward (*Khalifah*) on Earth?” “Will it bring me closer to my family’s well-being, or is it a fleeting desire?” This process aligns precisely with the definition of “mindful consumption” in the consumer psychology literature, which is understood as a conscious and intentional decision-making process focused on personal and societal well-being, rather than on the automatic, hedonistic pursuit of wants (Sheth et al., 2011; Gupta and Sheth, 2024). Accordingly, this orientation aligns with global trends in ethical consumerism, where mindfulness practices have been associated with choices toward sustainability and responsibility. This stands in contrast to impulse-driven consumption often catalyzed by modern marketing strategies that target subconscious desires. However, this effect is likely moderated by factors such as religiosity, socio-economic constraints, and cultural differences, which warrant empirical exploration in future studies.

The second mechanism is enhanced self-regulation (*Muraqabah*) and financial discipline. The constant state of self-observation fostered by *Muraqabah* is theorized to support executive self-regulatory capacities, which are associated with self-control and delayed gratification. While direct neuroscientific evidence specific to Islamic contemplative practices remains limited, this proposition is informed by findings from secular mindfulness research, which links attentional training to executive control processes (Zeidan et al., 2010). This orientation is further reinforced by explicit Islamic ethical injunctions against wastefulness (*Israf*) and opulent extravagance (*Itraf*). The Quran explicitly warns, “*Indeed, the wasteful are brothers of the devils*” (Abdel-Haleem, 2004, 17:27). Within this theoretical framework, the mindful individual may be better positioned to regulate spontaneous spending urges, adhere to predetermined budgets, and avoid detrimental financial practices, most notably interest-bearing debt (Riba), which is strictly prohibited in Islam (Azmat et al., 2021). This framework suggests the possibility of consumer orientations characterized by greater financial prudence and aversion to frivolous or conspicuous consumption, although such patterns are likely to vary across individuals and contexts. This presents a significant challenge to credit-based consumer economies and potentially an opportunity for businesses championing value, durability, and financial responsibility (Floren et al., 2020; Junaidi et al., 2022). Variation in practice adherence and external financial pressures may weaken or complicate these patterns, underscoring the need for empirical testing rather than assuming universality.

The third mechanism involves the expansion of ethical consideration in consumption. Mindfulness in Islam is not a solely inward-looking practice; it extends outward to the social and environmental consequences of one’s actions. A mind grounded in *Dhikr* and accountable through *Muraqabah* may become more attuned to a product’s lifecycle. The concept of *Halal* thus expands from a narrow focus on ritual slaughter to encompass a holistic view of ethicality (*Tayyib*). This includes concerns for animal welfare, fair labor practices in the supply chain, ethical sourcing of materials, and the environmental impact of production. This holistic ethical view also extends to financial practices, emphasizing transaction integrity (e.g., avoidance of *Riba*). Therefore, within this theoretical framework, brand authenticity, genuine corporate social responsibility (CSR), and transparent supply chains may function as salient drivers of brand preference and loyalty for some Muslim consumers. Trustworthiness (*Amanah*) may emerge as a highly salient attribute of a brand, as it reflects the integrity that some consumers strive to cultivate within themselves (Putra et al., 2023; Rana and Solaiman, 2023). A breach of this trust may be perceived not merely as poor business practice but as a violation of deeply held ethical principles. Nonetheless, the degree to which such ethical awareness translates into consistent purchasing behavior remains an open empirical question, as real-world consumption is often shaped by economic constraints, social norms, and market pressures.

### 3.2 Workplace well-being and performance

The influence of this cultivated psychological framework also extends into professional life, offering a foundation for enhanced well-being, focus, and ethical conduct in the workplace.

A primary benefit is the enhancement of cognitive focus and the reduction of reactivity. The daily practice of Salah requires, multiple times a day, a complete disengagement from work tasks to engage in an act of deep, focused attention. This structured interruption serves as a cognitive reset, pulling the individual out of the potentially mindless flow of emails and tasks and allowing for mental recalibration. From a theoretical perspective, this regular practice may support capacities related to sustained attention and cognitive control. Furthermore, the practice of Dhikr, silently repeating remembrances of God during work, can function as a continuous attentional anchor, helping to maintain focus on a single task and reducing susceptibility to the myriad distractions of the modern open-plan office. Perhaps more importantly, by fostering a non-judgmental awareness of arising thoughts and emotions [a core component of mindfulness defined by Thompson et al. (2011)], these practices provide the mental space to choose a response rather than being hijacked by an emotional reaction. This equips employees to handle stressful deadlines, critical feedback, or challenging interactions with colleagues with greater equanimity and deliberation. These mechanisms conceptually parallel findings from secular mindfulness neuroscience, where attentional training has been associated with prefrontal regulation and reduced amygdala reactivity, although direct neuroscientific evidence specific to Islamic contemplative practices remains limited. Studies indicate that prayer and mindfulness improve attention and emotional regulation in workplace settings (e.g., Wheeler et al., 2017). Drawing such parallels allows for cross-cultural dialogue in mindfulness science.

These practices also cultivate resilience (*Sabr*), encompassing patience, endurance, and self-restraint. In high-pressure contexts, Dhikr and remembrance offer a perspective that strengthens the ability to navigate setbacks. The constant awareness of a higher accountability (*Muraqabah*) also serves as a powerful reinforcement for ethical integrity and professional honesty. The understanding that one is ultimately accountable to a divine authority for one’s actions, including how one treats subordinates, manages company resources, and represents one’s organization, creates a powerful internal ethical compass. This orientation may reduce the likelihood of engaging in unethical behavior, cutting corners, or pursuing short-term gains at the expense of long-term integrity, thereby potentially contributing to cultures of trust within teams and organizations. Future intervention studies could directly test Dhikr- or Salah-based attentional resets as culturally adapted alternatives to MBSR, comparing outcomes in stress, focus, and ethical decision-making.

## 4 Discussion and synthesis: theoretical and practical implications

This conceptual study positions Islamic mindfulness not as a peripheral religious practice but as a culturally embedded cognitive-ethical system grounded in daily practices such as Muraqabah, Salah, Dhikr, and Niyyah. These practices are theorized to cultivate a psychological profile marked by intentionality, self-regulation, and transcendental accountability, which provides the conceptual foundation for the theoretical and practical implications discussed below.

### 4.1 Theoretical contributions to mindfulness literature

Islamic mindfulness enriches ongoing debates in mindfulness science by demonstrating how faith-based practices shape attention, emotional regulation, and ethical orientation. Overlaps with mainstream models include attentional anchoring (*Salah*, *Dhikr*) and emotional regulation (*Sabr*), while distinctive contributions include transcendental accountability and structured intentionality (*Niyyah*). These dimensions expand the boundaries of mindfulness theory beyond secular frameworks, offering support for a Culturally Embedded Mindfulness Model (CEMM). Integrating spirituality, ethics, and self-regulation, the Islamic model underscores that mindfulness is not universally uniform but deeply situated within cultural and theological contexts.

While related to constructs such as religiosity, spiritual coping, intrinsic religious orientation, and faith-based self-regulation, Islamic mindfulness is conceptually distinct. Rather than capturing belief strength, motivational commitment, or coping responses, it focuses on moment-to-moment attentional awareness, intentionality (Niyyah), and ongoing moral self-monitoring (Muraqabah) embedded in everyday practice. This process-oriented emphasis clarifies how religious meaning is cognitively enacted in daily decision-making, thereby distinguishing Islamic mindfulness as an explanatory construct rather than a re-labeling of adjacent concepts.

### 4.2 Implications for consumer behavior and marketing

From a consumer perspective, Islamic mindfulness is theorized to orient individuals toward more deliberate consumption, financial discipline, and heightened ethical awareness. This orientation may counter tendencies toward impulse-driven and credit-based consumption by emphasizing necessity, stewardship, and ethical sourcing.

For marketers and businesses, these insights highlight the importance of engaging authentically with Muslim consumers. Strategies may benefit from emphasizing integrity (Amanah), ethical supply chains, and community impact, rather than relying on superficial Halal certification or tokenized campaigns. Authenticity remains central, as branding perceived as insincere or opportunistic may risk misalignment with values shaped by intentionality and spiritual accountability.

### 4.3 Implications for workplace and leadership

In organizational settings, Islamic mindfulness may support employee focus, resilience, and ethical conduct. Practices such as prayer breaks can function as attentional resets, while *Dhikr* provides ongoing cognitive anchoring during routine tasks. Together, these practices may cultivate equanimity and may help reduce reactivity to workplace stressors.

Beyond productivity, sustained awareness of transcendental accountability may reinforces professional honesty and reduce the likelihood of unethical shortcuts, thereby potentially fostering trust within teams. For leaders, accommodating prayer and reflection extends beyond religious accommodation and can be understood as an investment in employee well-being and organizational integrity. Embedding transparency, ethical responsibility, and resilience into workplace culture aligns closely with the values fostered through Islamic mindfulness.

### 4.4 Synthesis

Taken together, the preceding sections demonstrate how Islamic mindfulness contributes to mindfulness theory while offering distinct insights for consumer behavior and workplace well-being. By situating mindfulness within a cultural religious system, this study illustrates the limits of exclusively secular models and highlights the value of culturally embedded traditions for expanding theoretical understanding and informing context-sensitive organizational and market practices. Although the Culturally Embedded Mindfulness Model (CEMM) is presented in a sequential form for conceptual clarity, the relationships it depicts are not assumed to be strictly linear or deterministic; rather, mindfulness practices, psychological mechanisms, and behavioral experiences are likely to interact in recursive and context-dependent ways, shaped by situational and cultural moderators over time.

## 5 Limitations and avenues for future research

As a conceptual paper, this work provides a theoretical framework that requires robust empirical validation. Its primary limitation is reliance on synthesizing adjacent literatures rather than presenting new data. Accordingly, the framework should be understood as exploratory, serving to clarify concepts and propose relationships rather than to establish causal effects. This makes it valuable in setting an agenda for future psychological and cross-cultural research on mindfulness across multiple disciplines.

In addition, this conceptual framework is subject to several interpretive limitations. Islamic mindfulness is characterized by substantial cultural, theological, and jurisprudential heterogeneity, and practices such as *Salah*, *Dhikr*, and *Niyyah* may vary in emphasis and enactment across regions, denominations (e.g., Sunni and Shia), and socio-cultural contexts. Moreover, research linking religious values to consumption and workplace behavior is susceptible to social desirability bias, as individuals may overstate alignment between normative religious ideals and actual practices. Finally, there is a risk of essentializing Muslim identity if Islamic mindfulness is interpreted as producing uniform psychological or behavioral outcomes; to mitigate this risk, future research should therefore adopt context-sensitive and empirically grounded approaches that account for intra-group diversity.

Future research should validate these insights through psychometric tools, intervention studies (e.g., *Salah* or *Dhikr*-based attentional resets), and comparative designs across cultures and religions. Longitudinal and mixed-method approaches may further strengthen understanding of how Islamic mindfulness practices evolve over time, while ethnographic work can complement these approaches by capturing lived experience.

## 6 Conclusion

Islamic mindfulness (*Muraqabah*, *Salah*, *Dhikr*, *Niyyah*) extends beyond spiritual practice to function as a culturally embedded cognitive and ethical system shaping orientation toward consumption and workplace behavior. By cultivating intentionality, disciplined self-regulation, and transcendental accountability, Islamic mindfulness may contribute to consumer and employee orientations characterized by greater deliberation, resilience, and ethical awareness.

This framework advances mindfulness scholarship by demonstrating how culturally embedded traditions enrich global theory and practice. It moves the discussion beyond narrow markers such as Halal certification to the deeper psychological and spiritual drivers of Muslim life without reducing religious practice to instrumental or utilitarian outcomes.

Finally, this paper offers Islamic mindfulness as a case study in culturally embedded mindfulness, emphasizing that mindfulness is neither universal nor culture-free. By foregrounding cultural and theological specificity, we call for future empirical research to validate, refine, and expand this framework, ensuring that global mindfulness discourse reflects both shared human capacities and meaningful cultural diversity.

## Publihser’s note

All claims expressed in this article are solely those of the authors and do not necessarily represent those of their affiliated organizations, or those of the publisher, the editors and the reviewers. Any product that may be evaluated in this article, or claim that may be made by its manufacturer, is not guaranteed or endorsed by the publisher.
